# Effect of 3 vs. 3 Soccer Small-Sided Game on Various Performance, Inflammatory, Muscle Damage and Hormonal Indicators in Semi-Professional Players

**DOI:** 10.3390/sports10070102

**Published:** 2022-06-28

**Authors:** Evangelos Bekris, Dimitrios I. Bourdas, Eleftherios Mylonis, Ioannis Ispirlidis, Emmanouil D. Zacharakis, Athanasios Katis

**Affiliations:** 1 School Physical Education and Sport Science, National and Kapodistrian University of Athens, Ethnikis Antistasis 41, 17237 Dafni, Greece; vagbekris@phed.uoa.gr (E.B.); elefmylonis@gmail.com (E.M.); 2 Section of Sport Medicine & Biology of Exercise, School of Physical Education and Sports Science, National and Kapodistrian University of Athens, 41 Ethnikis Antistasis, 17237 Athens, Greece; dbourdas@phed.uoa.gr; 3 School of Physical Education and Sport Science, Democritus University of Thrace, Panepistimioupoli, 69100 Komotini, Greece; iispyrli@phyed.duth.gr; 4 School of Physical Education and Sport Sciences of Serres, Aristotle University of Thessaloniki, Monastiriou 114, 53100 Florina, Greece; akatis@phed-sr.auth.gr

**Keywords:** aerobic-anaerobic training, exercise intensity, football, recovery, external load, internal load, reliability

## Abstract

The purpose of this study was to examine the effect of a soccer small-sided game (SSG) on performance, inflammatory, muscle damage and hormonal indicators. Twenty-two male soccer players participated and were assigned to either experimental (EXP = 12) or control (CON = 10) groups. Subjective fatigue (RPE) and lactate (La¯) were measured during the SSG; vertical squat jump (SJ), 20-m sprint, creatine kinase (CK), interleukin-6 (IL-6), cortisol (C), and testosterone (T) were measured before (PRE), after (POST), 24 h, 48 h, and 72 h after the SSG in the EXP group. The heart rate during the SSG reached 92 ± 3% of their HRmax, whereas La¯ and RPE reached 13.02 ± 1.60 mmol·L^−1^ and 15 ± 1 after SSG, respectively. The IL-6, different among measurements (F (1.04, 11.50) = 504.82, *p* < 0.001), peaked (3.52 ± 0.43 pg·mL^−1^ [95%CI; 3.28–3.77]) after the SSG and returned to baseline 24 h later. The CK, different among measurements (F (1.76, 19.32) = 93.96, *p* < 0.001), peaked (536.58 ± 124.73U·L^−1^ [95%CI; 466.01–607.15]) 24 h after the SSG and remained significantly higher than PRE condition in POST and up to 72 h later. The T/C ratio, significantly different among measurements (F (1.73, 19.05) = 12.12, *p* < 0.001), was at its lowest (0.44 ± 0.16 [95%CI; 0.35–0.54]) immediately after the SSG (*p* < 0.05) and returned to baseline after 24 h. It seems that 48 h (at the most) after an SSG is adequate time for players to recover, and a high training load should be avoided sooner than 24 h after an SSG.

## 1. Introduction

Soccer small-sided games (SSGs), where fewer players participate in reduced pitch size compared to official games (two teams of 11 players each compete on a pitch of ~100 × 60 m), are modified versions of the original game, constitute a very common and popular training practice, [1,2] and give coaches the option to use alternative forms of training and coaching. Modifying the number of players and the playing space in SSGs sets the desired participation time of each player, and a wide range of players’ technical, tactical, and physical skills can be improved [1,3].

In SSGs, the larger the dimensions of the field, the greater the distance traveled by the players [4,5]. In smaller fields (e.g., 3 vs. 3 compared to 6–10 vs. 6–10), the players’ acceleration and deceleration actions appear to increase in number [3,5,6]. Moreover, when the number of players in SSGs decreases (e.g., 1–3 vs. 1–3, compared to 4–6 vs. 4–6), there is usually an increase in heart rate (HR) [7,8]. Indeed, during SSGs with more players, the HR ranged from 80% to 86% of maximum HR (HRmax), and these SSG formats are considered suitable for high-intensity aerobic exercise [9]. In contrast, during SSGs with fewer players, HR ranges from 85% to 95% of HRmax and these SSG formats are considered suitable for anaerobic training [9]. In essence, the 3 vs. 3 SSG format allows us, on the one hand, to have high-intensity training (approaching 90–95% of HRmax and 8.4–9.1 mmol/L of lactate (La¯) concentration) [10,11] and, on the other hand, to apply various basic soccer tactics.

High-intensity physical activities, however, can increase the chances of muscle damage and cause inflammation responses [12]. So, the assessment of the physiological changes induced during both training and game is of great importance and, in modern soccer, several hormonal, biochemical and hematological indices are usually monitored in order to evaluate players’ performance and possible pathologies [13].

Changes in creatine kinase (CK) are associated with parameters such as overreaching, length, and intensity of training [14]. Creatine kinase is also considered a reliable enzyme for assessing muscle damage [15]. After intense exercise [16,17] and when increased eccentric load is observed, e.g., deceleration actions which commonly take place during a 3 vs. 3 SSG format, increased interleukin-6 (IL-6) is reported [5]. So, plasma IL-6 concentration is extensively used as a modulator of the inflammatory response [18]. Cortisol (C) and testosterone (T) have also been suggested as reliable indicators of training stress and fatigue [14]. The ratio of testosterone to cortisol (T/C), in particular, indicates the balance between anabolic and catabolic processes [19,20] and can provide important information on performance and recovery processes.

Although several earlier studies have examined the effects of a soccer game on the aforementioned indicators [20,21], examination of the effects of various SSG formats on these indicators has not received the appropriate attention. The evaluation of these indicators in different SSG formats is of great importance to soccer practitioners so as to apply strategies for improving physical performance and recovery after training, which may prevent overreaching and overtraining during the weekly training microcycle. Therefore, the purpose of the present study was to examine the effects of a 3 vs. 3 SSG training format on time course changes in sprint and jump performance, inflammatory, muscle damage, and hormonal indicators on three consecutive days after the SSG and, consequently, to assess the time players take to recover. The main hypothesis tested was that the SSG training protocol would have an effect on soccer players’ CK, IL-6, C, T, and T/C indicators.

## 2. Materials and Methods

### 2.1. Subjects

**Figure 1:**
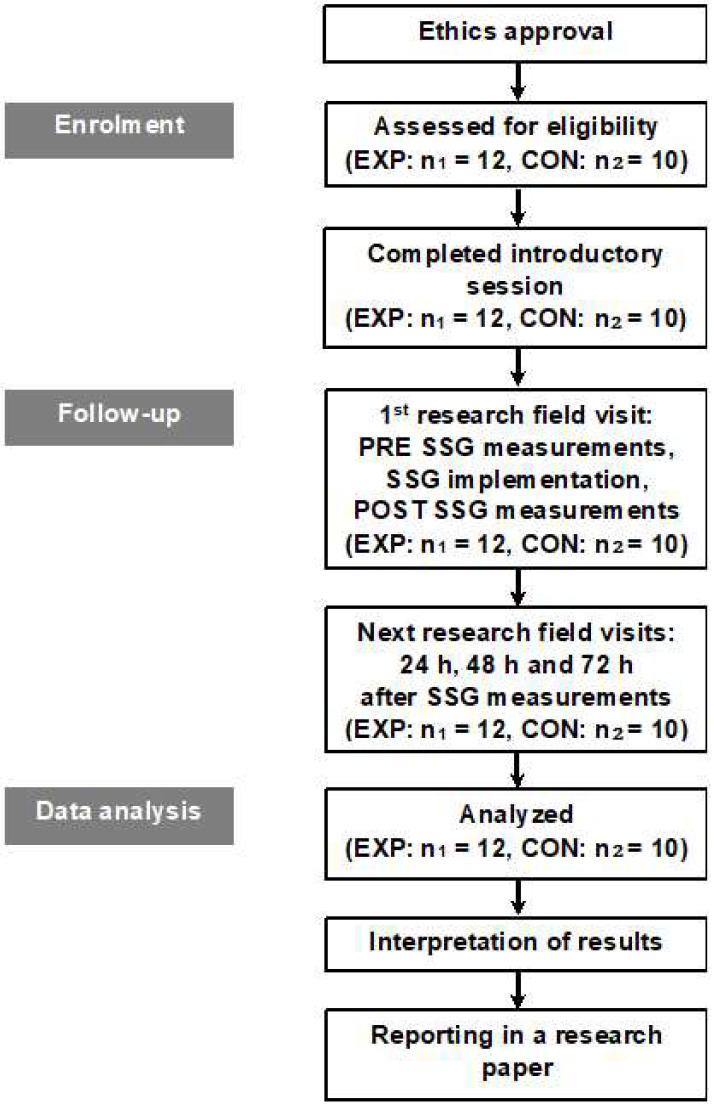
Flowchart of the experimental design. Abbreviations: CON, control group who did not engage in any type of strenuous physical activity; EXP, experimental group who implemented a small-sided game training format; SSG, soccer 3 vs. 3 small-sided game which included 8 sets (3 min duration and 3 min rest between the sets); POST, after small-sided game training format; PRE, prior to small-sided game training format.

The Ethics Committee of the University gave its institutional approval for the study. The study design is depicted in Figure 1. All potential participants filled out medical, physical activity, and smoking–sleeping habits questionnaires prior to the study [22,23,24,25]. Participants were chosen based on the following inclusion criteria: male, highly physically active (>1000 MET-min/week), non-smokers sleeping well (≥7 h·day^−1^), soccer players, over the age of 18, no history of neurological diseases or musculoskeletal abnormalities, not on medication or supplementary nutrition for the previous six months, and no participation in other sports activities during the study. Twenty-two male semi-professional soccer players participated in the current study and were randomly assigned to either experimental (EXP: n_1_ = 12) or control (CON: n_2_ = 10) groups (counterbalanced for players’ position) using a Latin-square design. The players’ anthropometric characteristics are presented in Table 1. The players competed in the 3rd division of the Greek National Soccer League. Their standard training program during the competitive period involved five training sessions per week (90 min per training session), in addition to one official game. All players were well aware of the aims, procedures, and risks involved in the study, and a written consent form was obtained from them before participation, based on the Declaration of Helsinki [26]. They were free to withdraw from the study with reason or no reason.

### 2.2. Introductory Session

#### 2.2.1. Anthropometric Characteristics

A digital scale (seca 880 weight scale, Seca Ltd., Hamburg, Germany) was used to measure participants’ nude body mass and an anastometer (seca 213 portable stadiometer, Seca Ltd., Hamburg, Germany) to measure their height. Body fat percentage was calculated using sex–age–Caucasian-specific formulae [27] after the thickness of seven skinfolds (chest, axilla, triceps, subscapular, abdomen, supra-iliac, and thigh) was measured (Harpenden Skinfold Caliper; Baty International, West Sussex, UK).

#### 2.2.2. Maximum Heart Rate and Yo-Yo Intermittent Recovery Test Level 1

Each player’s HRmax (Polar Sport tester, Polar Electro Oy, Kempele, Finland) was determined using the Yo-Yo Intermittent Recovery Test Level 1 (YYIRTL1) [28,29,30] and used as a reference value for the quantification of HR values observed during the SSG. The YYIRTL1 consisted of 20 m shuttle runs performed at continuously increased speeds until exhaustion [28,29,30]; these performance data are presented in Table 1.

### 2.3. Experimental Procedure

Five days after the introductory session (neither group was involved in any strenuous physical activity in the meantime), several indicators were measured before (PRE), immediately after (POST), 24 h, 48 h, and 72 h after the SSG format training (three-a-side game situation) in the EXP group. The same data were acquired from the CON group, which did not engage in any type of SSG. The EXP group consisted of 4 subgroups (×3 players, randomly counterbalanced for players’ position). Each (random) pair of subgroups was assigned to play a single SSG with a one-day difference between them (two in total). Regardless of the group’s assignment, all players were told to give it their all and that no feedback would be offered until the completion of the study. The EXP group was unaware that the CON group would not play an SSG, and the CON group was unaware that the EXP group would play an SSG. Both the players and the assessment researchers had no idea what the study’s true aims were (double-blind design).

The importance of maintaining regular sleeping habits, a pre-described (by the researchers) balanced diet (i.e., 50–60% carbohydrates energy intake, 25–30% fat, and 15% protein) for one week before and three days after the SSG, and the physical activities prior to all upcoming measurements was emphasized to the subjects in considerable detail. Participants arrived at the research field (sea level) at 7:00 a.m. after 10 h of fasting; immediately after baseline blood sample (i.e., 1.5 h prior to the SSG), a light standardized meal to control for micronutrients, selenium intake, and vitamins was ingested by the players. All participants were in generally good health during the study. All instruments and devices were calibrated according to the manufacturer’s requirements before each test.

#### 2.3.1. SSG Training Format

The SSG training format was conducted (9:00 a.m.) one week after the regular competitive season was finished (early May). The players did not have any severe training loads this week (just practiced regular game tactics and team cohesion) and did not take any supplements with an ergogenic or synergistic impact [31,32,33]. One day before the main protocol, participants were not involved in any training session.

The pitch size was 20 m × 25 m [10]. The SSG training format had an overall duration of 45 min (8 sets × 3 min with 3 min active recovery between sets) in order to simulate the half of a realistic soccer game and was conducted with no goalkeepers and free touches. Keeping ball position was the aim of the game. Coach encouragement using standardized instructions and ad libitum water ingestion were allowed [10]. Air temperature ranged from 23 to 26 °C and humidity from 55 to 65%.

#### 2.3.2. Heart Rate, Subjective Fatigue and Lactate Measurements

During the SSG, HR was recorded every 5 s using short-range radio telemetry (Polar Sport tester, Polar Electro Oy, Kempele, Finland). The subjective rate of perceived exertion (RPE) was recorded at rest prior to the SSG (PRE), at the end of the 2nd, 5th, and 8th set (POST) of the SSG and at 24 h, 48 h, and 72 h after the SSG, using the 6–20 linear Borg scale [34]. A portable blood analyzer (Lactate Plus- Nova Biomedica, Waltham, MA, USA) was used for the measurement of the blood La^−^ concentration. The La^−^ measurement was performed by touch strip to capillary blood drop (5–25 μL) from the left index finger. Blood La^−^ concentration was measured 1-min before the SSG (PRE), 1-min after the end of the 2nd, the 5th and 8–10 min after the end of the 8th set (POST) of the SSG. Lactate concentration, HR and RPE were used as SSG’s intensity indicators. The RPE and all following measurements performed 24 h, 48 h, and 72 h after the SSG were measured at 10:00 a.m. (after overnight fasting) to avoid any chronobiological effect.

#### 2.3.3. Jumping Test

Forty-five minutes prior to the SSG (PRE) and after a standardized 5 min warm-up [60–70% intensity on a leg cycle ergometer (894E, Monark, Varberg, Sweden) except after the SSG], the participants performed three maximum squat jumps (SJ) with arm swing, with a 30 s recovery between each trial [35]. All SJs were performed on a customized uniaxial force plate (OptoJump System, Microgate, Bolzano, Italy). The platform uses a strain gauge (Model LC4204-K600; A&D Co. Ltd., Tokyo, Japan) capable of measuring vertical ground reaction force and contains photocells at a distance of 2 mm from the ground, which are constantly communicating. The device detects any interruptions in communication between the bars and calculates their duration. Thus, it was possible to assess the vertical jump. The best SJ based on maximum height was used for further analysis. The SJ test was repeated 15 min after the SSG (POST) and 24 h, 48 h, and 72 h after the SSG.

#### 2.3.4. Sprint Test

Five minutes after each SJ test, the participants performed three maximum 20-m sprints with a 30 s recovery between each sprint. Sprint times were recorded using infrared photoelectric cells interfaced to a timing system (Saint Wien Digital Timer Press H5K, Lu-Chou City, Taipei Hsien, Taiwan) with a time resolution of 0.01 s and a measurement error of ±0.01 s. The best trial was used for further analysis.

#### 2.3.5. Hematological Measurements

Ten ml of blood were collected by venipuncture from the forearm vein, stored in tubes with a gel separator, and transported to the laboratory the same day to be examined under a constant temperature (23–25 °C). A blood sample was centrifuged for 5 min at 3200 rpm for CK analysis, and the serum recovered was examined using Biochemistry 3000 BT Plus^®^ kit with Beckman Coulter^®^ (Biotecnica Instruments S.p.H. Rome, Italy; Beckman Coulter International S.A., Nyon, Switzerland). The measurement of IL-6 was made on a standard ELISA reader (Spark 10M; Tecan, Mannedorf, Switzerland) by an ELISA kit (R & D Systems Inc., Minneapolis, MN, USA) according to the manufacturer’s instructions. For T and C analysis, the chemiluminescence procedure was used following the Bio System Kit specifications (Elecsys 2010, Roche Diagnostics (Hellas) S.A. Maroussi, Greece) as referenced elsewhere [36,37]. Blood samples were collected 1.5 h prior to the SSG (PRE), immediately after the SSG (POST), and 24 h, 48 h, and 72 h after the SSG, and were analyzed in duplicate.

### 2.4. Statistical Analysis

All results are presented as mean (M) ± standard deviation (SD) [95% confidence interval (CI)]. The Levene’s and Shapiro–Wilk tests were used to determine the homogeneity and normality of the acquired data. For comparisons in anthropometric and dependent variables in rest condition (PRE) between groups, independent *t*-tests were applied. A 2 by 4 (Groups × Time) mixed analysis of variance (ANOVA) with repeated measures on the time factor was used to analyze changes between groups across multiple time measurements for La^−^, a 2 by 5 (Groups × Time) for 20-m sprint, SJ, IL-6, CK, C, T, and T/C, a 2 by 7 (Groups × Time) for RPE and a 2 by 9 (Groups × Time) for HR. When the assumption of sphericity was violated, the degrees of freedom (df) for main effects, interactions, and error terms were adjusted according to Greenhouse–Geisser ε. Significant interactions were followed up with post hoc analysis of simple effects and analytical pairwise comparisons with Bonferroni correction to determine significant differences [38] between time measurements for the experimental group. The effect sizes were calculated using partial eta squared *(η*_p_^2^). Statistical analysis was performed in SPSS version 21.0 (SPSS, Inc., Chicago, IL, USA), and statistical significance was set at *p* < 0.05. The experimental sample size of 12 (≥11) was established in statistical power calculations (80%) to detect statistical significance (GraphPad StatMate Version 2.0, GraphPad Software Inc., La Jolla, CA, USA) for the IL-6, CK, and C variables based on our ~0.75 pilot effect size [observed after an SSG training format implementation (3 vs. 3) between experimental (n_1_ = 6) and control groups (n_2_ = 3) using the same experimental design and revealing significant changes).

## 3. Results

In all dependent variables, our statistical analysis showed that (i) there were no significant differences in PRE conditions between CON and EXP groups (*p* > 0.05) and (ii) time measurements for the CON group did not reach statistical significance (*p* > 0.05), as was expected. For those reasons, statistical conclusions for significant interactions are based on simple effects (F-values with corrected df) and Bonferroni pairwise comparisons across time measurements for the EXP group in all measured variables [38,39].

**Figure 2:**
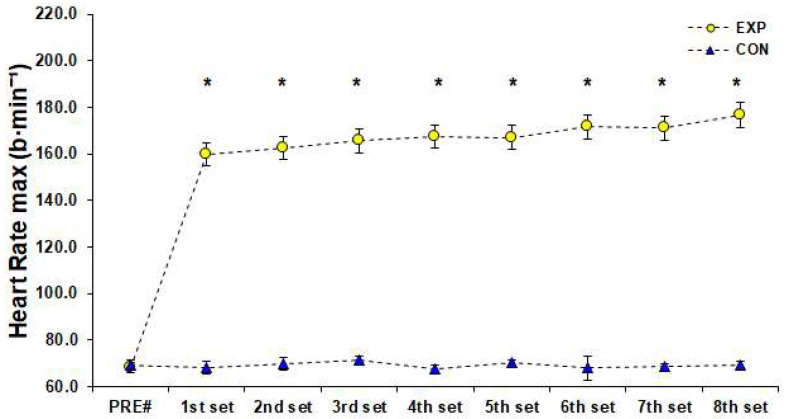
Heart rate maximum values before (PRE) and during (1st–8th set) of a small-sided game (3 vs. 3), (M ± SD). * Significant difference from PRE measurements in experimental group (*p* < 0.05). # References to rest values. Abbreviations: CON, control group who did not engage in any type of strenuous physical activity; EXP, experimental group; M, mean value; SD, standard deviation.

**Figure 3:**
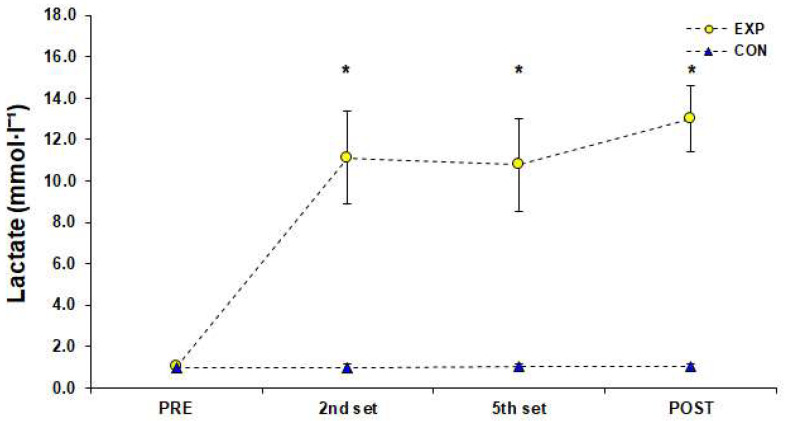
Lactate concentration values before (PRE), during (2nd and 5th set), and after (POST) a small-sided game (3 vs. 3), (M ± SD). * Significant difference from PRE measurements in experimental group (*p* < 0.05). Abbreviations: CON, control group who did not engage in any type of strenuous physical activity; EXP, experimental group; M, mean value; SD, standard deviation.

**Figure 4:**
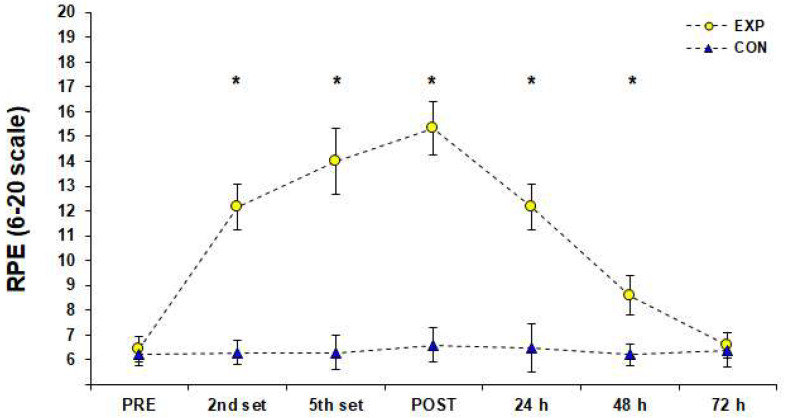
Subjective rate perceived exertion values before (PRE), during (2nd and 5th set), after (POST) and for three consecutive days after a small-sided game (3 vs. 3), (M ± SD). * Significant difference from PRE measurements in experimental group (*p* < 0.05). Abbreviations: CON, control group who did not engage in any type of strenuous physical activity; EXP, experimental group; M, mean value; RPE, rate perceived exertion; SD, standard deviation.

**Figure 5:**
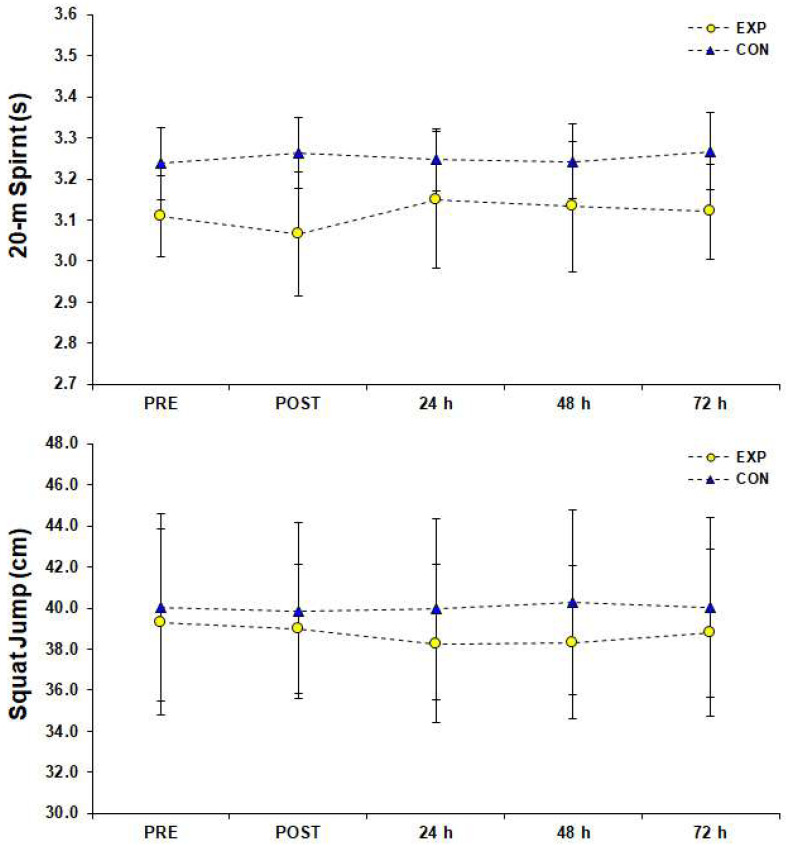
Values of performance assessments (top: 20-m sprint, bottom: squat jump) before (PRE), after (POST) and for three consecutive days after a small-sided game (3 vs. 3), (M ± SD). Abbreviations: CON, control group who did not engage in any type of strenuous physical activity; EXP, experimental group; M, mean value; SD, standard deviation.

The HR during the 3 vs. 3 SSG training format was 168 ± 7 b·min^−1^ [95%CI; 166–169] corresponding to 87 ± 4% of HRmax achieved in YYIRTL1 (84 ± 3%, 85 ± 3%, 86 ± 3%, 87 ± 3%, 87 ± 3%, 89 ± 3%, 89 ± 3%, 92 ± 3% after the end of the 1st–8th sets, respectively). There was a significant difference (F (1.00, 11.04) = 5647.93, *p* < 0.001, *η*_p_^2^ = 1.00) for HRmax measurements achieved in the SSG of the EXP group and Bonferroni pairwise comparisons indicated that HRmax values were gradually higher from rest to the end of the 8th set of the SSG training (Figure 2). Blood La^−^ concentration reached 11.13 ± 2.23 mmol·L^−1^ [95%CI; 9.87–12.39] after the end of the 2nd set, 10.79 ± 2.24 mmol·L^−1^ [95%CI; 9.87–12.39] after the end of the 5th set, and 13.02 ± 1.60 mmol·L^−1^ [95%CI; 12.12–13.93] after the end of the 8th set of the SSG training format. The simple effects analysis for blood La^−^ indicated a significant difference among measurements (F (1.81, 19.97) = 44.06, *p* < 0.001, *η*_p_^2^ = 0.80), and pairwise comparisons showed that La¯ was significantly higher than rest values (PRE) after the 2nd and 5th set of the SSG, whereas it peaked after the end of the SSG (Figure 3). Statistically significant changes (F (3.43, 37.76) = 292.98, *p* < 0.001, *η*_p_^2^ = 0.96) were found among RPE estimations of the EXP group. Pairwise comparisons showed that RPE increased significantly (progressively), peaked immediately after the SSG (*p* < 0.05), and returned to baseline 72 h after the SSG (Figure 4). The results of simple effects analysis in SJ and 20-m sprint measurements for the EXP group did not show statistically significant differences (F (1.68, 18.45) = 1.40, *p* ≥ 0.05, *η*_p_^2^ = 0.11 and F (2.02, 22.20) = 2.65, *p* ≥ 0.05, *η*_p_^2^ = 0.19 respectively; Figure 5).

**Figure 6:**
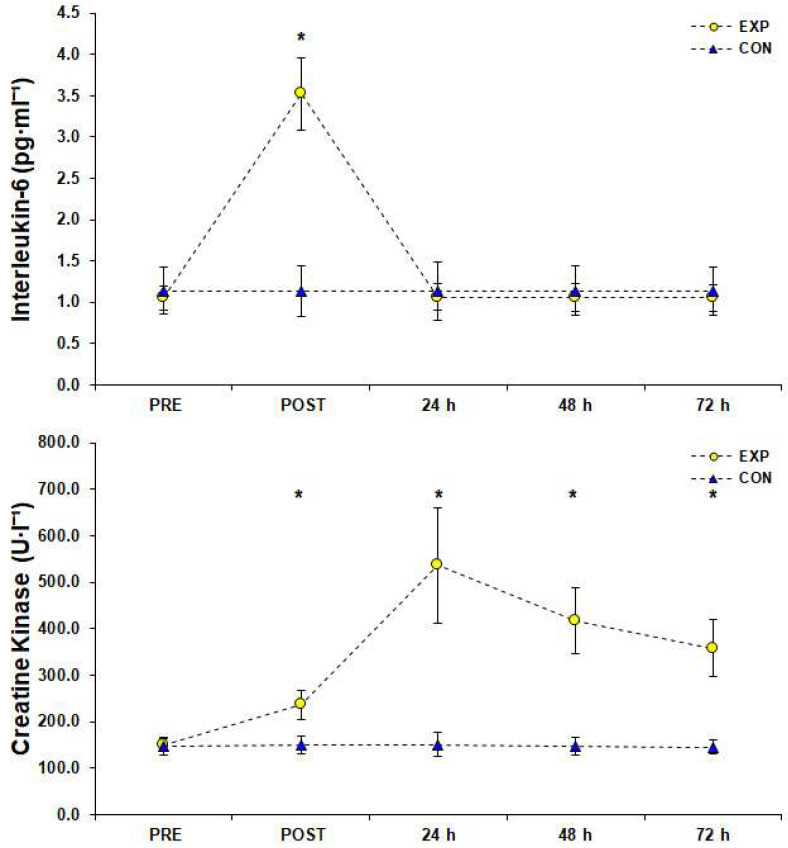
Inflammatory responses and muscle damage measurements (top: interleukin-6, bottom: creatine kinase) before (PRE), after (POST), and for three consecutive days after a small-sided game (3 vs. 3), (M ± SD). * Significant difference from PRE measurements in experimental group (*p* < 0.05). Abbreviations: CON, control group who did not engage in any type of strenuous physical activity; EXP, experimental group; M, mean value; SD, standard deviation.

The simple effects analysis in IL-6 responses showed a significant difference among measurements (F (1.04, 11.50) = 504.82, *p* < 0.001, *η*_p_^2^ = 0.98) and Bonferroni post hoc analysis showed that IL-6 reached its peak (3.52 ± 0.43 pg·mL^−1^ [95%CI; 3.28–3.77]) immediately after the SSG and returned to baseline 24 h later (*p* < 0.05, Figure 6). Statistical analysis of CK responses showed a significant difference among measurements (F (1.76, 19.32) = 93.96, *p* < 0.001, *η*_p_^2^ = 0.90) and post hoc analysis revealed that CK peaked (536.58 ± 124.73 U·L^−1^ [95%CI; 466.01–607.15]) 24 h after the SSG and remained significantly higher than PRE condition in POST, 48 h, and (even though ranged in normal values) 72 h after the SSG (*p* < 0.05, Figure 6).

**Figure 7:**
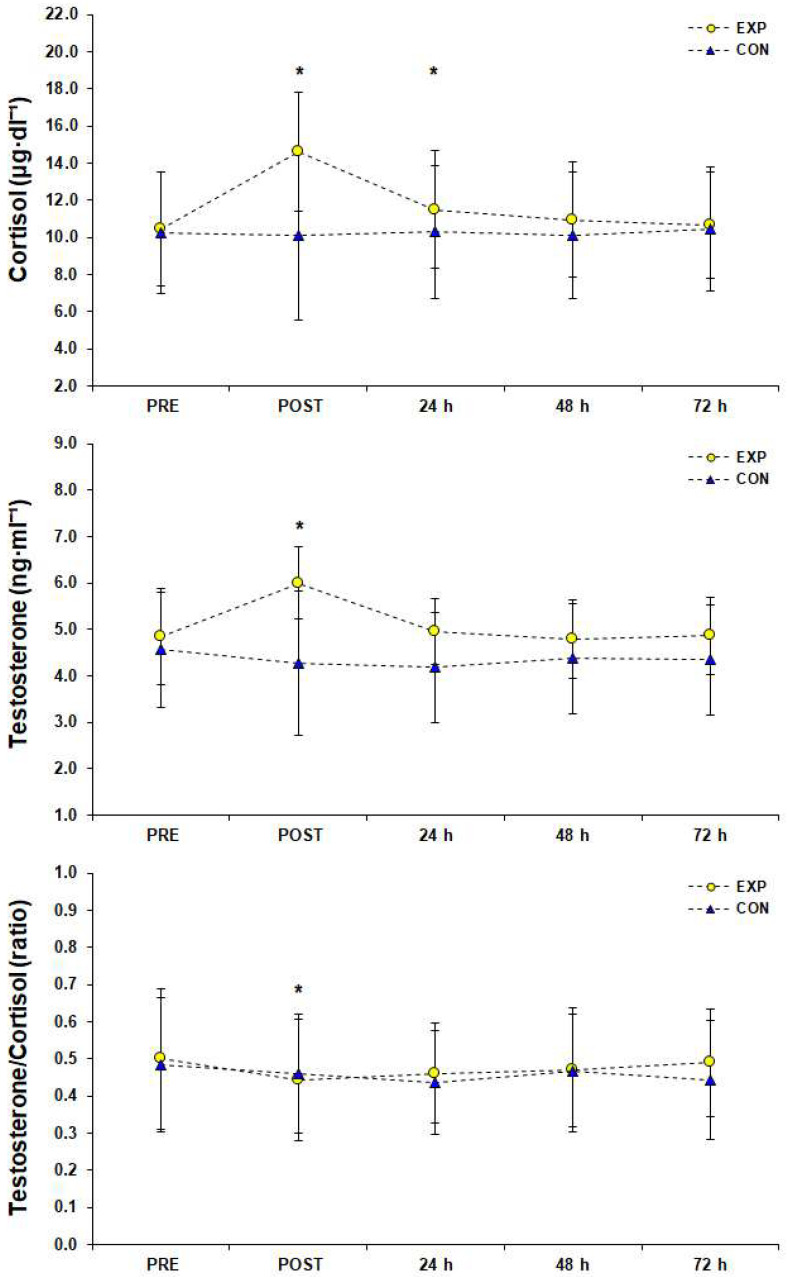
Hormonal responses (top: cortisol, middle: testosterone, bottom: testosterone to cortisol ratio) before (PRE), after (POST), and for three consecutive days after a small-sided game (3 vs. 3), (M ± SD). * Significant difference from PRE measurements in experimental group (*p* < 0.05). Abbreviations: CON, control group who did not engage in any type of strenuous physical activity; EXP, experimental group; M, mean value; SD, standard deviation.

The simple effects analysis in C responses showed a significant difference among measurements (F (1.00, 11.00) = 122.21, *p* < 0.001, *η*_p_^2^ = 0.92), whereas post hoc analysis showed that C peaked (14.62 ± 4.58 μg·dL^−1^ [95%CI; 12.03–17.21]) immediately after the SSG (*p* < 0.05) and remained significantly higher than PRE condition for 24 h after the SSG (*p* < 0.05) before gradually returning to baseline, 48–72 h after the SSG (Figure 7). Statistical analysis in T responses showed a significant difference among measurements (F (1.69, 18.62) = 47.39, *p* < 0.001, *η*_p_^2^ = 0.81). Post hoc analysis revealed that T peaked (6.00 ± 1.55 ng·mL^−1^ [95%CI; 5.13–6.88]) immediately after the SSG (*p* < 0.05) and returned to baseline 24 h later (Figure 7). Statistical significance (F (1.73, 19.05) = 12.12, *p* < 0.001, *η*_p_^2^ = 0.52) was found for T/C ratio of the EXP group among measurements and post hoc analysis showed that the lowest value of T/C ratio (0.44 ± 0.16 [95%CI; 0.35–0.54]) was observed immediately after the SSG (*p* < 0.05) and returned to baseline the next morning (Figure 7).

## 4. Discussion

Although a number of previous studies have examined the effects of a soccer game on performance and the physiological strain of the athletes [20,21], the effects of SSG formats on these metrics have not gotten the attention they deserve. The purpose of the current study was to investigate the effects of a 3 vs. 3 SSG training format on time course changes of various performance, inflammatory, muscle damage, and hormonal indicators in three consecutive days after an SSG in male semi-professional players. The main findings indicate that after implementing the SSG training format, performance indexes such as SJ and 20-m sprint were not impaired compared to rest values, inflammatory response peaked immediately after the SSG and returned to rest values in 24 h, CK concentration peaked 24 h after the SSG and remained significantly elevated for at least 72 h after the SSG, and T/C ratio recovered in 24 h.

During the SSG, the participants reached HR corresponding to >90% of their HRmax, RPE ~ 15–16 (95%CI), and their blood La¯ level reached ~ 12.12–13.93 mmol/L (95%CI) after the SSG (POST). All these observations indicate that the athletes’ physiological strain during the SSG reached the observed values of anaerobic training [9], meaning that our outcomes reflect the participants’ adjustments after regular high-intensity training [10,11]. However, the participants’ power and sprint performance remained similar to rest values after the SSG and in the next three days, whereas RPE return to rest values on the third day.

The exercise probably changes the level of anti-inflammatory and pre-inflammatory cytokines such as IL-6 [40]. Conversely, the increase of IL-6 is related to exercise intensity, duration, and muscle mass activation [41], which is why IL-6 is used as an indicator of post-exercise inflammation. It has been reported that IL-6 concentration peaked immediately after a soccer game and returned to initial levels 24 h after the game [42], whereas in another study, which used a 90-min exercise protocol with repetitive speeds, an increase in IL-6 occurred immediately after and 1 h after the exercise [43]. Consistent with these findings, the present study found that IL-6 levels followed the same time course indicating no inflammation response 24 h after the SSG training format.

Creatine kinase has been widely used as an indicator of muscle damage and muscle loading during exercise [12,44]. As reported by previous relevant studies, CK concentrations range 250–400 U·L^−1^ after a soccer game [21,42], values similar to the ones observed in the present study. Eccentric muscle contractions executed during decelerations, changes of running direction [45], and several activities with the ball commonly performed in SSGs are associated with increased CK concentration and potentially increased muscle damage [46,47]. Maximum CK concentration has been observed 24–48 h after the game, returning to rest values between 48 and 120 h after the game [48], which is in agreement with our observations.

Cortisol concentration increases after a soccer game [42] and usually returns to normal levels within 24 h [49]. It has been reported that increased levels of C are probably observed as a consequence of the psychological aspects of the official soccer game [50]. Haneishi et al. also found larger increases in C concentrations after a competitive soccer game as compared to a training session [49]. In the current study, C values peaked after the SSG and gradually returned to normal values 48 h after the SSG training. Therefore, it seems that the alteration of C concentration in the present study was similar to the one commonly observed after a soccer game.

The present results showed that although T values peaked after the SSG, T returns to rest values in 24 h. It has been reported that when catecholamines, such as adrenaline and noradrenaline, are not significantly increased, especially during friendly games or when no mental stress is observed, e.g., during SSGs as compared to official soccer games, there is no tendency for excessive testosterone production during and post exercise [51]. Therefore, the SSG training format applied in this study seems to have common features with a prolonged aerobic endurance exercise as far as T production is concerned [52], rather than with an exercise of high muscle demand, which includes a great number of sprints, changes of direction, accelerations, and decelerations, associated with force exercise mechanisms [50]. 

The T/C ratio is commonly used as an indicator of training load and is associated with players’ fatigue, exhaustion, and decreased performance [20]. Presently, it was observed that (except immediately after SSG training format) the T/C ratio was not significantly affected in a range of three days (Figure 7). If this is the case, then the current SSG training format has only had an acute term effect on physical stress and probably did not overexhaust the participants. So, bearing in mind all our results (i.e., gradual reduction of RPE and CK, no performance aspects impairment, and no increased inflammation response or reduced balance between the anabolic and catabolic processes 24–48 h after SSG training), participants seem to be physically ready for new training loads and actions the second day after the SSG; this information could be useful in designing appropriate training sessions.

### Strengths and Limitations

To the best of our knowledge, studies that have investigated the impact of a soccer 3 vs. 3 SSG on SJ, 20-m sprint performance and on IL-6, CK, T, C, and T/C response in 3 consecutive days after the SSG are very rare, especially concerning male semi-professional soccer players. However, the activity pattern (e.g., number of sprints, accelerations, and decelerations) and distances covered by each player in relation to the athletes’ positions are not documented, which really is a limitation of the present study. Moreover, the results of the present study should be interpreted with caution since there is no direct comparison to responses following an 11 vs. 11 full-scale soccer match. Likewise, this study’s generalized conclusions are confined to adult male semi-professional soccer players, with characteristics similar to those presented in Table 1, and refer to the current SSG training format characteristics. Consequently, the findings of this study will need to be validated by future studies for different SSG training formats, different soccer categories, and female players; additionally, more hematological variables and performance metrics should be investigated, and the possible interaction of the athletes’ position, SSG covered distance, and physical fitness level should be taken into account.

## 5. Conclusions

Observations derived from the present study on male semi-professional players revealed that 3 vs. 3 SSG training format did not reduce SJ and 20-m sprint performance, whereas 24 h after SSG training, the induced inflammatory response was significantly diminished, RPE and CK concentration were gradually reduced compared to peak values, and T/C ratio returned to rest values. Moreover, CK, C, and T values from the second day after the SSG onwards lay within the normal range for healthy adults [15,53,54]. Based on this evidence, a 48-h period (at the most) after an SSG seems to be an adequate time for players to recover and should be taken into consideration when designing soccer SSG training programs. Moreover, a high training load should be avoided earlier than 24 h after an SSG; therefore, in order to reduce the risk of overtraining or injury, team practitioners should adapt the weekly training workload (intensity and volume) as needed, ensuring adequate post-SSG recovery while minimizing the risk of training interruption [55]. Future studies, which would also examine the players’ technical and tactical characteristics in different SSG formats, including physical performance and hormone production, should provide important information to coaches and could prove to be useful tools in designing soccer training programs.

## Figures and Tables

**Table 1:** Participants’ anthropometric characteristics values (M ± SD [95%CI]).

	EXP	CON
Age (year)	22.42 ± 3.96 [20.17–24.66]	22.20 ± 4.02 [19.71–24.70]
TP (year)	6.08 ± 3.42 [4.15–8.02]	6.00 ± 3.60 [3.77–8.22]
H (cm)	182.41 ± 5.52 [179.28–185.53]	180.15 ± 6.54 [176.10–184.20]
BM (kg)	† 82.68 ± 7.54 [78.41–86.95]	80.23 ± 7.18 [75.78–84.68]
BM (kg)	‡ 82.53 ± 7.55 [78.26–86.80]	-
BF (%)	† 15.31 ± 5.01 [12.48–18.15]	13.04 ± 3.53 [10.85–15.23]
ADC (m) #	2400.00 ± 574.87 [2074.74–2725.26]	2464.00 ± 612.52 [2084.36–2843.64]
S (km·h^−1^) #	17.29 ± 0.96 [16.75–17.84]	17.40 ± 1.02 [16.77–18.03]
HRmax (b·min^−1^) #	196 ± 6 [193–199]	195 ± 5 [192–199]
HR (b·min^−1^)	† 68 ± 2 [67–70]	69 ± 2 [68–70]

† prior to small-sided game (3 vs. 3). ‡ after small-sided game (3 vs. 3). # at Yo-Yo Intermittent Recovery Test Level 1 (YYIRTL1). Abbreviations: ADC, accumulated distance covered at YYIRTL1; BF = body fat; BM = body mass; CON, control group (n_2_ = 10) who did not engage in any type of strenuous physical activity; EXP, Experimental group (n_1_ = 12) who implemented a small-sided game training format; H, height; HR, heart rate at rest; HRmax, heart rate maximum; M, mean value; S, maximum speed at YYIRTL1; SD, standard deviation; TP, soccer training period; 95%CI, 95% confidence interval.

## Data Availability

The corresponding author will consider written reasonable requests for data sharing.
